# Daily-Life Social Experiences as a Potential Mediator of the Relationship Between Parenting and Psychopathology in Adolescence

**DOI:** 10.3389/fpsyt.2021.697127

**Published:** 2021-08-05

**Authors:** Robin Achterhof, Olivia J. Kirtley, Maude Schneider, Ginette Lafit, Noëmi Hagemann, Karlijn S. F. M. Hermans, Anu P. Hiekkaranta, Aleksandra Lecei, Inez Myin-Germeys

**Affiliations:** ^1^Research Group Psychiatry, Department of Neurosciences, Center for Contextual Psychiatry, KU Leuven, Leuven, Belgium; ^2^Clinical Psychology Unit for Intellectual and Developmental Disabilities, Faculty of Psychology and Educational Sciences, University of Geneva, Geneva, Switzerland; ^3^Research Group on Quantitative Psychology and Individual Differences, Department of Psychology, KU Leuven, Leuven, Belgium; ^4^Research Group Adapted Physical Activity and Psychomotor Rehabilitation, Department of Rehabilitation Sciences, KU Leuven, Leuven, Belgium; ^5^Research Group Psychiatry, Department of Neurosciences, Center for Clinical Psychiatry, KU Leuven, Leuven, Belgium

**Keywords:** parenting, adolescence, psychopathology, social interaction, experience sampling method, parenting style, daily life, social interactions

## Abstract

Adolescence is a vulnerable period for psychopathology development, and certain parenting styles are consistent and robust predictors of a broad range of mental health outcomes. The mechanisms through which maladaptive parenting styles affect the development of psychopathology are assumed to be largely social in nature. Yet, the social mechanisms linking parenting to psychopathology are unexplored at arguably the most important level of functioning: daily life. This study aims to identify the associations between three parenting styles, and the experience of daily-life social interactions. Furthermore, we aim to explore the extent to which these parenting styles and altered daily-life social experiences are associated with psychopathology. In this study, we recruited a sample of *N* = 1,913 adolescents (63.3% girls; mean age = 13.7, age range = 11 to 20) as part of the first wave of the longitudinal cohort study “SIGMA”. Parenting styles (psychological control, responsiveness, and autonomy support) and psychopathology symptoms were assessed using a retrospective questionnaire battery. The experienced quality of social interactions in different types of company was assessed using the experience sampling method, ten times per day for 6 days. Direct associations between parenting styles and general quality of daily-life social experiences were tested using a three-level linear model, revealing significant associations between social experiences and different parenting styles. When interaction effects were added to this model, we found that maternal responsiveness and paternal psychological control mainly related to altered qualities of social interactions with parents, while paternal autonomy support was associated with better experiences of non-family social interactions. Finally, an exploratory path analysis highlighted how both paternal autonomy support and altered quality of non-family interactions are uniquely associated with psychopathology levels. These findings demonstrate the general and pervasive effects of maladaptive parenting styles, as parenting seems to broadly affect adolescents' interactions with different types of social partners in everyday life. Moreover, they illustrate a potential mediated relationship in which altered daily-life social interactions could drive the development of psychopathology. A stronger focus may be required on the role of altered day-to-day social experiences in the prevention and potentially, the treatment, of adolescent psychopathology.

## Introduction

Mental health problems are strongly shaped by the specific manners in which children have been raised, as certain parenting dimensions are strong and robust predictors of a broad range of psychopathological symptoms. For example, concurrent and longitudinal evidence has emphasized the maladaptive effects on child and adolescent development of parental psychological control, which is characterized by control that undermines children's emotional experience and expression and involves, e.g., love withdrawal and guilt induction (1). On the other hand, more positive parenting has been described, for example, in terms of parental support or responsiveness, which represents parents' sensitive responses to situations when a child is distressed (2). Parental autonomy support is another type of parenting assumed to be positively related to children's adjustment, and is characterized by, e.g., parents' allowing their children to make their own choices, and acknowledging children's own perspective (3). As such, these positive parenting dimensions are hypothesized to be associated with more adaptive psychosocial development. Ample previous research has highlighted how parental psychological control is a unique risk factor for the development of internalizing psychopathology (4–8), while parental responsiveness and autonomy support are protective against the development of psychiatric symptoms (7, 9, 10).

Understanding the lasting impact of parenting styles on children's development is particularly relevant during adolescence, which represents the period where most psychopathology develops (11). Adolescence is also a period of significant social change, when individuals develop independence as they move away from parents and toward peers (12, 13). As adolescents start interacting more and more with non-family members, however, those with experiences of adaptive parenting styles are expected to be best-equipped to engage with others. An adolescent who has been supported by their parents in the development of their autonomy is expected to feel comfortable seeking out this autonomy in their relations with others (14), and adolescents who have experienced responsive parenting can be expected to enter the social arena with a greater sense of security (2). Conversely, experiences of psychologically controlling parenting are thought to instill a sense of insecurity and negative self-perception, which, in turn, can be expected to spill over into social interactions with others (8, 14). As such, parenting styles help lay the foundation for how adolescents interact with other people as they venture into the world.

Healthy and positive social interactions with both family and non-family members are, in turn, considered as important protective factors for maintaining good mental health. Alternatively, when the described parent-driven socialization goes awry, psychopathology may develop. Fundamentally, the established link between parenting and psychopathology can be considered largely social in nature: parenting more generally affects adolescents' social interactions (15) which, when they are consistently negative, can contribute to the development of mental health problems. However, while the relationship between parenting and psychopathology symptoms is often assumed to be mediated by altered social interactions, this has—to our knowledge—never been comprehensively explored in daily life.

Social correlates of parenting and psychopathology are usually assessed using retrospective self-report questionnaires. Although research that employs retrospective measures can be valuable for assessing general processes, it fails to consider several important aspects of social processes. Naturalistic social interactions are dynamic, context-dependent, fleeting, subtle, involve all senses, and as such, are difficult to capture outside of the real world (16, 17). An alternative method that does allow for the capturing of social interactions in daily life is the Experience Sampling Method (ESM), also referred to as Ecological Momentary Assessment or EMA (18–20). ESM is an intensive longitudinal method in which participants are prompted multiple times per day to report on their momentary experiences, thoughts, feelings, and context as they go about their day-to-day lives. In assessing social processes in context, ESM allows for capturing both the relatively objective characteristics of day-to-day social interactions (i.e., who are you interacting with, and where?) and the associated subjective experience (i.e., how do you feel about the person you're with?).

To some extent, research employing ESM to study social processes has already identified how daily well-being and the quality of daily social interactions are linked to people's parenting experiences (15, 21–23), while there is also increasing evidence for the relationship between psychopathology symptoms and altered experiences of social interactions (22–25). Interestingly, these studies indicate how, generally, both parenting experiences and psychopathology relate more to an altered *quality* of social interactions (e.g., feeling belonging to current company; preferring to be alone when with others) rather than to changes in the *quantity* of social behaviors. These findings suggest that the mediating role of social processes in the relationship between parenting and psychopathology can be reliably assessed at the level of daily life, and that this mediating role is likely determined more by altered subjective social experiences than by differences in social behaviors. However, as the results of the studies pertained to all social interactions that participants reported on, these results lack some necessary specificity. Participants in ESM studies engage with different people throughout the sampling period—both with family and with non-family members. Parenting styles are likely differentially associated with social experiences when the associated social interactions pertain to family vs. non-family members. As discussed, to truly understand the impact of parenting on adolescents' socialization, it is imperative to specifically assess how parenting relates to the qualities of social interactions when adolescents interact with people outside of the family.

Therefore, in this study, we aim to test the associations between the parenting styles of psychological control, autonomy support, and responsiveness, and altered daily-life social experiences; and to investigate the specificity of the effects of maternal/paternal parenting styles on daily-life social interactions when participants are with parents vs. when with non-family members. Moreover, in examining these relationships, we will also consider differences in age and gender. As adolescents get older and achieve more independence, the relationship between parenting and psychopathology may weaken. At the same time, previous research has suggested how parenting styles are more strongly associated with psychopathology when they refer to the same-sex parent (26). Accordingly, we will also investigate whether the investigated relationships differ across genders and as a function of age.

Our registered hypotheses are as follows (see registration or Supplemental Material for full hypotheses as registered):

1. *Perceived parental (maternal and paternal)****responsiveness****is associated with a more****positive****social experience in daily life, when also accounting for the proportion of time spent with mother/father*.
2. *Perceived parental (maternal and paternal)****psychological control****is associated with a more****negative****social experience in daily life, when also accounting for the proportion of time spent with mother/father*.
3. *Perceived parental (maternal and paternal)****autonomy support****is associated with a more****negative****social experience in daily life, when also accounting for the proportion of time spent with mother/father*.
4. *Perceived****maternal responsiveness****is associated with more****positive****experiences of****mother interactions****in daily life, when also accounting for the effects of maternal autonomy support, maternal psychological control, and paternal parenting styles*.
  *4.1 This association is stronger than the association between maternal responsiveness and social experience when with non-family members*.
5. *Perceived****maternal psychological control****is associated with a more****negative****social experience****of mother interactions****in daily life, when also accounting for the effects of maternal autonomy support, maternal responsiveness, and paternal parenting styles*.
  *5.1 This association is stronger than the association maternal psychological control and social experience when with non-family members*.
6. *Perceived****maternal autonomy support****is associated with a more****positive****social experience****of mother interactions****in daily life, when also accounting for the effects of maternal psychological control, maternal responsiveness, and paternal parenting styles*.
  *6.1 This association is stronger than the association between maternal autonomy support and social experience when with non-family*.
7. *Perceived****paternal responsiveness****is associated with a more****positive****social experience****of father interactions****in daily life, when also accounting for the effects of paternal autonomy support, paternal psychological control, and maternal parenting styles*.
  *7.1 This association is stronger than the association between paternal responsiveness and social experience when with non-family members*.
8. *Perceived****paternal psychological control****is associated with a more****negative****social experience****of father interactions****in daily life, when also accounting for the effects of paternal autonomy support, paternal responsiveness, and maternal parenting styles*.
  *8.1 This association is stronger than the association between paternal psychological control and social experience when with non-family members*.
9. *Perceived****paternal autonomy support****is associated with a more****positive****social experience****of father interactions****in daily life, when also accounting for the effects of maternal psychological control, maternal responsiveness, and paternal parenting styles*.
  *9.1 This association is stronger than the association between paternal autonomy support and social experience when with non-family members*.
10. *Perceived****maternal and paternal responsiveness****are uniquely associated with a more****positive****social experience of interactions with****non-family members****, when also accounting for the effects of the effect of maternal/paternal responsiveness and autonomy support*.
11. *Perceived****maternal and paternal psychological control****are uniquely associated with a more****negative****social experience of interactions with****non-family members****, when also accounting for the effects of maternal/paternal responsiveness and autonomy support*.
12. *Perceived****maternal and paternal autonomy support****are uniquely associated with a more****positive****social experience of interactions with****non-family members****, when also accounting for the effects of the effect of maternal/paternal responsiveness and psychological control*.
13. *All reported effects are stronger for younger participants*
14. *All reported maternal effects are stronger for female participants; all reported paternal effects are stronger for male participants*.

Responsiveness and autonomy support are hypothesized to have positive associations with the experience of social interactions, while for psychological control, we expect negative associations. In addition to testing these hypotheses, we conduct exploratory analyses using a comprehensive path model, where general psychopathology levels are associated with both parenting styles and mean social experiences when in company of mother/father/non-family, and where these social experiences in turn are associated with different parenting styles. Although studies with cross-sectional data preclude claims about the temporal sequence of events underlying mediation effects (27, 28), they can be used to illustrate the contemporaneous associations that might form the basis for longitudinal mediation, which can be investigated in future longitudinal research.

## Materials and Methods

### Participants

A sample of *N* = 1,913 adolescents were recruited and tested between January 2018 and June 2019 as part of the first wave of the longitudinal cohort study SIGMA (29). Participants were recruited for the SIGMA study through one of 22 participating secondary schools in the Flanders region in Belgium. Potential participants were briefed about the content of the study before they could voluntarily sign up, with permission of at least one parent or caregiver. There were no specific in- or exclusion criteria for this study, apart from the ability to read and understand Dutch.

At the time of testing, all participants were either in the first (roughly aged 12/13), third (aged 14/15), or fifth grade (aged 16/17) of the Flemish secondary education system. As the Flemish school system allows students to repeat school years, and because students who immigrated to Flanders may enter a grade at an older age, a small number of participants included in this study are over 18 (eight 19-year-olds; one 20-year-old). In line with modern definitions of adolescence (30), we retained these participants in the full sample.

No information on ethnic or geographic background, or on racial identity was collected. Asking about ethnic groups or racial identity specifically is relatively uncommon in Belgium, and to our knowledge, no standard ethnicity or racial identity questions/response categories exist in Dutch (within Belgium/the Netherlands). Participants were asked whether they identified with any non-Belgian country, and 186 (26.1% of 713 responses to this question) responded with identifying with at least one country other than Belgium.

### Procedure

The SIGMA study consisted of two main parts: Retrospective questionnaires and daily-life measurements. For the first part of the study, participants were administered a self-report questionnaire battery in one 100-min session that they were asked to complete on a provided tablet computer in their own classroom. These questionnaires included, among others, those on parenting style and psychopathology that are used in the current study, but also questionnaires on social support, bullying, trauma, and other factors [for full questionnaire battery, see (29)]. Each participant was asked to start completing the questionnaires at a different specific part of the questionnaire battery—thereby ensuring comparable missing-ness patterns across all questionnaires. When participants did not want to answer any specific item, they were given the option of answering ‘*I do not wish to answer this question'*.

At the end of this initial 100-min session, participants were instructed about the second, daily-life part of the study. Participants were provided with a smartphone pre-installed with the MobileQ application (31), through which they would receive the ESM questionnaires for the following 6 days. Participants were instructed to go about their daily lives as usual throughout the ESM period, and to answer the random prompts whenever they were notified. They were also guided through the questionnaire by one of the researchers, to ensure that the content of every item was clear.

Each day in the ESM period, at semi-random times between 7.30 a.m. and 10.30 p.m., participants received ten notifications on the provided smartphone asking them to complete a 45-item questionnaire on their mood, thoughts, behaviors, and context (see Supplementary Material for full ESM list). The semi-randomness of this ESM design refers to the prompts being distributed at random times within each of ten 90-min blocks. There was at least 15 min between consecutive prompts, participants had 90 s to respond to each prompt, and participants had 90 s to complete each individual item in the questionnaire. For all participants, the ESM period involved four school days and 2 weekend days. For ESM prompts sent out during class time, participants were given permission by the school and teachers to fill out these daily questionnaires in the classroom. After returning the study material at the end of the ESM period, participants were rewarded with a 10-euro gift voucher.

### Measures

#### Retrospective Questionnaires

##### Parenting Styles

Questionnaires on parenting styles were adapted from three of the four subscales of the aggregated ‘*General Parenting Style'* measure, which have been previously used in Dutch-speaking adolescent samples (32). Each participant was asked to complete a maternal and paternal version of the subscales for the most important mother and father figures in their lives. If they indicated having no mother or father figure, participants were instructed to skip those respective subscales.

Parental psychological control (e.g., ‘*My mother/father brings up my past mistakes whenever she/he criticizes me'*) was measured using 8 items from the Psychological Control Scale—Youth Self-Report (1). Parental responsiveness (e.g., ‘*My mother/father can make me feel better when I am upset'*) was measured using 7 items from the Child Report of Parent Behavior Inventory (33, 34). Parental autonomy support (e.g., ‘*My mother/father lets me choose what to do, whenever that is possible'*) was measured using 7 items from the Autonomy Support Scale of the Perceptions of Parents Scale (35).

##### Psychopathology

Psychopathology was assessed using the Dutch translation of the 53-item Brief Symptom Inventory (BSI-53), which has been validated for use in adolescent and adult populations (36–38). The BSI-53 consists of nine subscales on specific past-week psychiatric symptomatology, including somatization, obsessiveness, insecurity in social contact, depressiveness, anxiety, aggression and hostility, phobic anxiety, paranoid thinking, and psychoticism symptoms, plus four additional items. Participants were presented with a list of problems of each of these subscales, and were then asked to indicate to what extent, if at all, they had been bothered by each problem throughout the past week (including the day of testing). All items were then scored ranging from ‘*0 Not at all'* to ‘*4 Extremely'*. The specific subscale scores were not used in the current study. Instead, as per the BSI-53 manual, a Global Severity Index (GSI) score was calculated by taking the mean across all items. As such, the GSI represents the general presence and severity of psychopathology symptoms, ranging from 0 to 4.

Psychopathology is relatively undifferentiated during adolescence (39)—and this is also reflected in the high inter-correlations between psychopathology dimensions that we found in earlier factor analysis on psychopathology symptoms in the adolescents of this sample (22). In this study, we assess psychopathology levels from a broad spectrum of psychiatric complaints, as an indicator of general psychological distress. The GSI represents all items included in the BSI-53. Previous psychometric investigations of the BSI-53 in adolescents have suggested that it assesses a valid and primarily unidimensional construct of general psychological distress (37, 38).

#### Experience Sampling

The following items from our daily ESM questionnaire were used to construct all relevant moment-level variables (note that the full ESM questionnaire is listed in the Supplementary Material): ‘*Who am I with?*' (non-mutually exclusive answer options: ‘*father*', ‘*mother*', ‘*other family (from nuclear family)*', ‘*other family (outside of nuclear family)*', ‘*friend(s)*', ‘*other peers*', ‘*teacher*', ‘*other (familiar) people*', ‘*unfamiliar people*', ‘*no-one*'; if participants indicated to be in company, they were also presented with the following four social experience items ‘*I feel at ease in this company*', ‘*I feel appreciated by this company*', ‘*I feel like I belong*', and ‘*I would rather be alone*' (answer options here ranged from ‘*1. Not at all*' to ‘*7. Very much*').

Using the company information, a momentary ‘*company type*' variable was computed with answer options ‘*with mother*' (i.e., when in the company of mother but no one else), ‘*with father*' (i.e., when in the company of father but no one else), ‘*with non-family*' (i.e., when in the company of any [combination of] non-family members), and ‘*mixed social situations*' (i.e., all other social situations). Only the first three categories of this variable were used to assess the differential social experience when with different people, as we did not have any specific hypothesis for the ‘mixed' social situations.

Using this information, the variables ‘*proportion of social interactions with mother*' and ‘*proportion of social interactions with father*' were constructed by computing the per-person proportion of time spent with either mother or father across all completed ESM questionnaires. These variables indicate the time spent with mother and father per participant and are used as covariates in subsequent analysis.

As main outcome variable, a mean momentary ‘*social experience*' variable was computed, by taking the average of the four momentary social experience variables (whereby the item ‘*I would rather be alone*' was reversed). As registered prior to data analysis, we first assessed whether the internal consistency of these variables was sufficient (i.e., between-person Cronbach's alpha > 0.50)—and it was, at 0.75. This variable was also used to compute the three person-level means of ‘*mean social experience when with mother*', ‘*mean social experience when with father*', and ‘*mean social experience when with non-family members*'—all of which were used as variables in the comprehensive path model.

### Statistical Analyses

The analyses for this study are 2-fold: First, we tested the specific associations between parenting styles and momentary social experiences using linear multilevel models—both overall, and for different types of company. Second, we explored the possible mediating effects of daily-life social experience in the relationship between parenting styles and general psychopathology a path analysis. In this path analysis, we did not consider the multilevel nature of the data, as—to the best of our knowledge—traditional multilevel mediation models do not allow for the testing of the multiple Level 2 Level 1 Level 2 paths that are included in the model (40).

The major R packages that we used were tidyverse (v.1.3.0) (41) for data manipulation, nlme (v. 3.1-150) (42), and lavaan (v. 0.6-7) (43) for analyses, tidySEM (v. 0.1.8) (44) and ggplot2 (v. 3.3.3) (45) for visualizations, and knitr (v. 1.30) (46) for producing analysis reports.

#### Multilevel Models Predicting Social Experience From Parenting Styles

To test the effects of parenting style on overall social experience, a three-level linear analysis was conducted using the ‘*nlme'*-package in R, with moments nested within participants, participants nested in schools, and with random intercepts but fixed slopes. In each model, the time-variant (i.e., moment-level) ‘*social experience*' variable was predicted by the six time-invariant (i.e., person-level) parenting style variables. Also included in this analysis were the time-variant ‘*company*' factor variable (without the ‘*mixed company*' category), and the time-invariant covariates of age, gender, and the ‘*proportion of social interactions with mother*' and ‘*proportion of social interactions with father*' variables. Then, to test whether the effect of parental bonding on social experience is different for different types of company, the same multilevel model was tested whereby a number of interaction terms were added simultaneously. These terms included the interaction effects between age and each parenting style, between gender and each parenting style, and between company type and each parenting style. To identify the nature of possible interaction effects, we visualized the estimated social experience in different companies for different levels of the specific parenting style (i.e., −1/+1 standard deviation of the mean).

#### Exploratory Comprehensive Path Model Predicting Psychopathology From Both Parenting Styles and Mean Social Experiences

For the path model, we estimated one comprehensive path model with all six parenting styles as predictors of the three mean social experience variables (i.e., when with mother, father, and non-family) and of psychopathology. Psychopathology was also separately predicted by the three mean social experience variables in this model. This path model is tested using the ‘*lavaan'*-package in R.

### Power Analysis

In the registration for this study, we described the power analysis that we would perform, following a strategy described by Lafit et al. (47). Power was computed by performing the confirmatory analyses described above on 1000 Monte Carlo-based simulations, for a three-level model with 10 beeps per day for 6 days and an average compliance rate of 50%, aiming to achieve 0.80 power. For each of the 1,000 simulated samples, the power is then estimated as the number of Monte Carlo replicates in which the null hypothesis is rejected.

Parameter estimates to construct the simulated data sets were based on similar data from an adolescent and young adult twin data set described elsewhere (i.e., TwinssCan) (48, 49). Access to this data set only allowed us to test the power of the direct associations between parenting styles and mean daily-life social experience, as participants were not asked in the experience sampling whether they were with mothers/fathers specifically—thereby not allowing for the testing of our described interaction effects. Also, in this TwinssCan data set, all variables were defined differently than, as parenting styles were based on the Parental Bonding Instrument (50), and mean social experience was constructed using slightly different items. In addition, this power analysis did not include the third school level in the multilevel analysis, as participants were not clustered in schools in the TwinssCan data set as they were in the SIGMA data set.

The results of this power analysis revealed >0.99 power for the positive associations between maternal/paternal responsiveness and mean social experience, and very low power (between 0.05 and 0.10) for the associations between the other parenting styles and mean social experience. Note, however, that these latter associations and its accompanying effect size estimates (between psychological control/autonomy support and social experience) had been very weak and non-significant in the estimation of these parameters in the TwinssCan data set. Although power was therefore extremely low for these associations, we still decided to continue with the analyses as planned, as (1) the differentially constructed variables in the TwinssCan might have produced biased parameter estimates, and (2) interaction effects might still be significant, even though main effects are not. Full code for both the parameter estimates and for the power analysis can be found on the OSF-page for this project.

### Open Science Practices

All hypotheses and analyses were registered following data collection, but prior to data access, i.e., a post-registration (51). When performing all analyses for this study, as planned in our registration (main registration available on OSF-page here: https://bit.ly/3wH3LPE; and the supplement that includes the exploratory mediation analysis here: https://bit.ly/2R9Cvsu), we encountered some inconsistencies across research questions, hypotheses and analysis plan, and some suboptimal analytic decisions. Therefore, we decided to change some details of the analysis, and report these deviations in full in the transparent-changes document (see Supplementary Material). Also, all code and output of the confirmatory, exploratory and sensitivity analyses have been uploaded to the OSF page for this study (https://bit.ly/3x39NtZ). All ESM items are publicly available in the ESM Item Repository (www.esmitemrepository.com) (52).

## Results

Descriptive information of the sample is presented in Table 1. For *n* = 114 participants, no ESM data was available, and these were excluded from the current analyses, thus leaving an initial sample size of *n* = 1,799. A more extensive description of this study and the sample's characteristics is included elsewhere (29).

**Table 1:** Descriptives of included variables.

	**Variable**	**Available *n***	**Mean (SD)**	**Median**	**Range**
Demographics	Age	1,789	13.7 (1.8)	13.0	11.0–20.0
	Gender (% females)	1,785	63.7		
	Number of completed beeps (out of 60)	1799	24.8 (12.7)	24.0	1.0–59.0
Psychopathology (BSI-53)	GSI	1,452	0.9 (0.6)	0.7	0.0–3.4
Social behavior (ESM)	% in company	1,799	86.4 (11.4)	91.3	0.0–100.0
	% time with mother only	1,799	5.1 (2.1)	0.0	0.0–100.0
	% time with father only	1,799	3.0 (1.7)	0.0	0.0–76.2
	% with non-family members	1,799	46.8 (5.8)	45.8	0.0–100.0
Social experiences (ESM)	With mother only	874	6.0 (1.3)	6.5	1.0–7.0
	With father only	610	5.3 (1.8)	6.1	1.0–7.0
	With non-family members	1,756	5.7 (1.0)	5.86	1.0–7.0
	Overall	1,792	5.8 (1.0)	6.0	1.17–7.00
Maternal parenting styles	Autonomy support	1,372	26.9 (4.9)	27.0	7.0–35.0
	Psychological control	1,361	15.2 (5.9)	14.0	8.0–38.0
	Responsiveness	1,393	30.6 (5.3)	32.0	7.0–35.0
Paternal parenting styles	Autonomy support	1,411	25.7 (5.0)	26.0	7.0–35.0
	Psychological control	1,385	15.8 (5.7)	15.0	8.0–40.0
	Responsiveness	1,433	27.6 (6.9)	29.0	7.0–35.0

Correlations between all parenting styles and the proportion of time spent with mother/father are presented in Table 2. The different parenting styles are moderately correlated with each other (between 0.21 and 0.71, and the parenting styles are only very weakly associated or not significantly associated with the proportion of time spent with mother or father (between 0.01 and 0.07).

**Table 2:** Correlations between the six parenting styles and the covariate of time spent with mother/father.

	**PPC**	**PR**	**MAS**	**MPC**	**MR**	**% with father only**	**% with mother only**
PAS	−0.54^**^	0.71^**^	0.45^**^	−0.29^**^	0.36^**^	0.06^*^	−0.07^**^
PPC		−0.49^**^	−0.21^**^	0.52^**^	−0.25^**^	−0.03	0.06^*^
PR			0.27^**^	−0.25^**^	0.41^**^	0.07^**^	−0.05
MAS				−0.52^**^	0.67^**^	−0.03	0.01
MPC					−0.52^**^	0.02	0.02
MR						−0.01	0.01
% with father only							0.05^*^

* *p < 0.05;1*

** *p < 0.01.*

*PAS, Paternal Autonomy Support; PPC, Paternal Psychological Control; PR, Paternal Responsiveness; MAS, Maternal Autonomy Support; MPC, Paternal Psychological Control; MR, Maternal Responsiveness*.

### Associations Between Parenting Styles and General Daily-Life Social Experiences

Our first research question referred to the prediction of mean daily-life social experience by parental autonomy support, psychological control, and responsiveness. Results pertaining to these hypotheses (Table 3) indicate how maternal responsiveness and paternal autonomy support are both significant positive predictors of more positively experienced social interactions in daily life; while maternal psychological control is a negative predictor of mean social experience.

**Table 3:** Predicting momentary social experience from parenting styles, and from the cross-level interactions between parenting styles and company types.

		**Model 1 (no interaction effects)**			**Model 2 (with interaction effects)**		
		***B (SE)***	***95% CI***	***p***	***B (SE)***	***95% CI***	***p***
	Intercept	5.71 (0.05)	5.61; 5.82	** <0.001**	5.69 (0.06)	5.58; 5.80	** <0.001**
Covariates	Gender (female)	−0.03 (0.06)	−0.14; 0.08	0.62	−0.03 (0.06)	−0.14; 0.07	0.54
	Age	−0.04 (0.02)	−0.07; −0.00	***0.042***	−0.04 (0.02)	−0.08; −0.01	***0.018***
	% time with father	−1.30 (0.43)	−2.14; −0.46	**0.003**	−1.36 (0.43)	−2.20; −0.52	**0.002**
	% time with mother	−0.45 (0.32)	−1.09; 0.18	0.16	−0.39 (0.32)	−1.03; 0.25	0.23
	Father company^*^	−0.15 (0.04)	−0.23; −0.08	** <0.001**	−0.17 (0.04)	−0.25; −0.09	** <0.001**
	Mother company^*^	0.43 (0.03)	0.37; 0.49	** <0.001**	0.42 (0.03)	0.36; 0.48	** <0.001**
Parenting style predictors	MAS	0.02 (0.01)	−0.00; 0.03	0.059	0.03 (0.02)	−0.00; 0.06	0.10
	MPC	−0.01 (0.01)	−0.03; −0.01	***0.028***	−0.01 (0.01)	−0.04; 0.01	0.34
	MR	0.02 (0.01)	0.00; 0.03	***0.025***	0.01 (0.01)	−0.02; 0.03	0.71
	PAS	0.03 (0.01)	0.01; 0.05	**0.001**	0.03 (0.02)	−0.01; 0.06	0.10
	PPC	−0.01 (0.01)	−0.03; 0.00	0.08	−0.02 (0.01)	−0.05; 0.00	0.07
	PR	0.01 (0.01)	−0.00; 0.02	0.06	0.01 (0.01)	−0.01; 0.03	0.38
Mother × Parenting style interaction effects	Mother company × MAS				0.01 (0.01)	−0.01; 0.03	0.21
	Mother company × MPC				−0.01 (0.01)	−0.03; 0.00	0.06
	Mother company × MR				0.02 (0.01)	0.00; 0.04	***0.028***
	Mother company × PAS				−0.03 (0.01)	−0.05; −0.01	**0.004**
	Mother company × PPC				−0.00 (0.01)	−0.02; 0.01	0.58
	Mother company × PR				0.00 (0.01)	−0.01; 0.02	0.69
Father × Parenting style interaction effects	Father company × MAS				−0.00 (0.01)	−0.03; 0.03	0.87
	Father company × MPC				0.02 (0.01)	−0.00; 0.04	0.12
	Father company × MR				−0.01 (0.01)	−0.04; 0.01	0.30
	Father company × PAS				−0.00 (0.01)	−0.03; 0.03	0.93
	Father company × PPC				−0.04 (0.01)	−0.07; −0.02	** <0.001**
	Father company × PR				0.00 (0.01)	−0.02; 0.02	0.67
Gender × Parenting style interaction effects	Gender × MAS				−0.02 (0.02)	−0.05; 0.02	0.39
	Gender × MPC				−0.00 (0.02)	−0.03; 0.03	0.96
	Gender × MR				0.02 (0.02)	−0.01; 0.06	0.16
	Gender × PAS				0.01 (0.02)	−0.03; 0.05	0.67
	Gender × PPC				0.02 (0.02)	−0.01; 0.05	0.15
	Gender × PR				0.00 (0.01)	−0.02; 0.03	0.80
Age × Parenting style interaction effects	Age × MAS				−0.00 (0.00)	−0.01; 0.01	0.95
	Age × MPC				0.00 (0.00)	−0.01; 0.01	0.66
	Age × MR				−0.01 (0.00)	−0.02; 0.00	0.13
	Age × PAS				0.00 (0.00)	−0.01; 0.01	0.43
	Age × PPC				−0.00 (0.00)	−0.01; 0.01	0.85
	Age × PR				−0.00 (0.00)	−0.01; 0.00	0.19

* *Within-person variable, compared to reference category: ‘Non-family member company'.*

*PAS, Paternal Autonomy Support; PPC, Paternal Psychological Control; PR, Paternal Responsiveness; MAS, Maternal Autonomy Support; MPC, Paternal Psychological Control; MR, Maternal Responsiveness; p-values in bold italics indicate p < 0.05; p-values in bold only indicate p < 0.01*.

While included as covariates (and thus not part of the confirmatory hypotheses), we note how the proportion of time spent with father (but not mother) is significantly and *negatively* associated with mean social experience. Also, compared to social interactions with non-family members, participants generally reported a more negative social experience when they interact with their father only, and a more positive social experience when interacting with their mother only.

### Associations Between Parenting Styles and Social Experience in Different Companies

In Table 3, the results of the models including the cross-level interaction effects between parenting style and different types of company are also presented. First, no direct associations between parenting style and social experience remained significant when including these interaction terms. Results indicated some significant cross-level interaction effects: Between maternal responsiveness and being with mother vs. non-family (positive interaction); between paternal autonomy support and being with mother vs. non-family (negative interaction); and between paternal psychological control and being with father vs. non-family (negative interaction).

**Figure 1:**
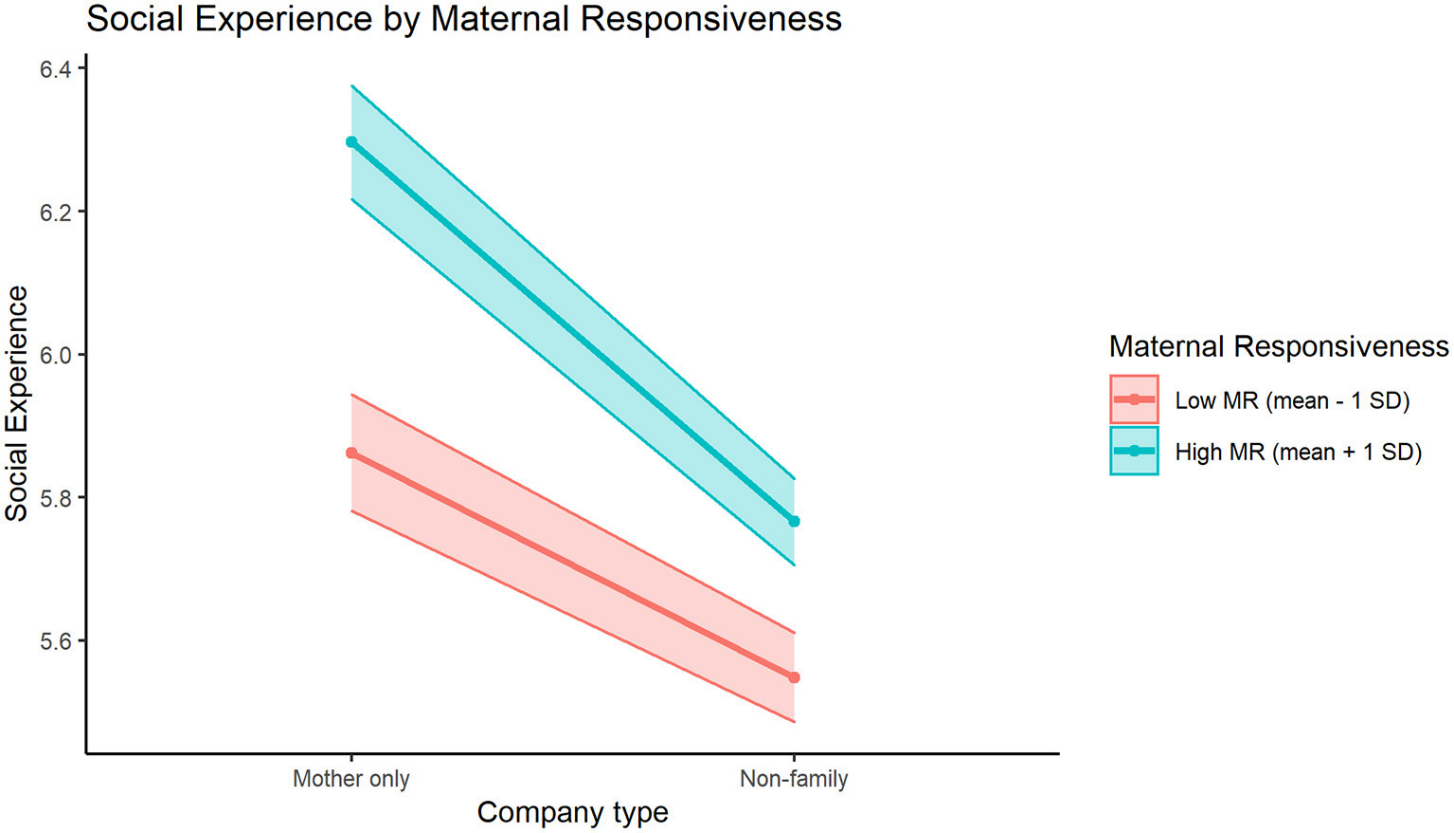
The significant interaction effect between MR (Maternal Responsiveness) and maternal vs. non-family company in the prediction of mean social experience, for participants ± 1 standard deviation of the Maternal Responsiveness mean (5.27).

**Figure 2:**
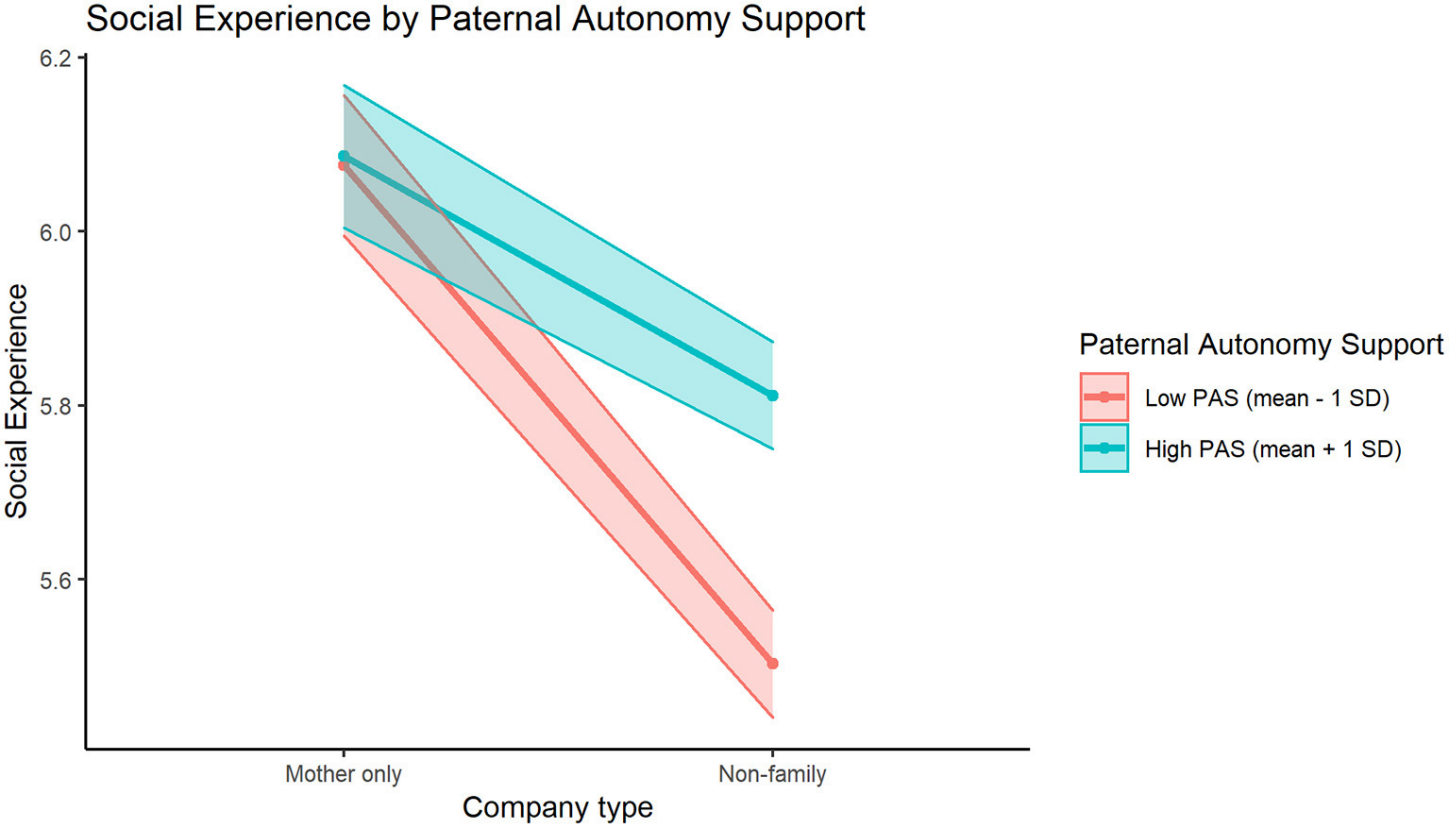
The significant interaction effect between PAS (Paternal Autonomy Support) and maternal vs. non-family company in the prediction of mean social experience, for participants ±1 standard deviation of the Paternal Autonomy Support mean (4.95).

**Figure 3:**
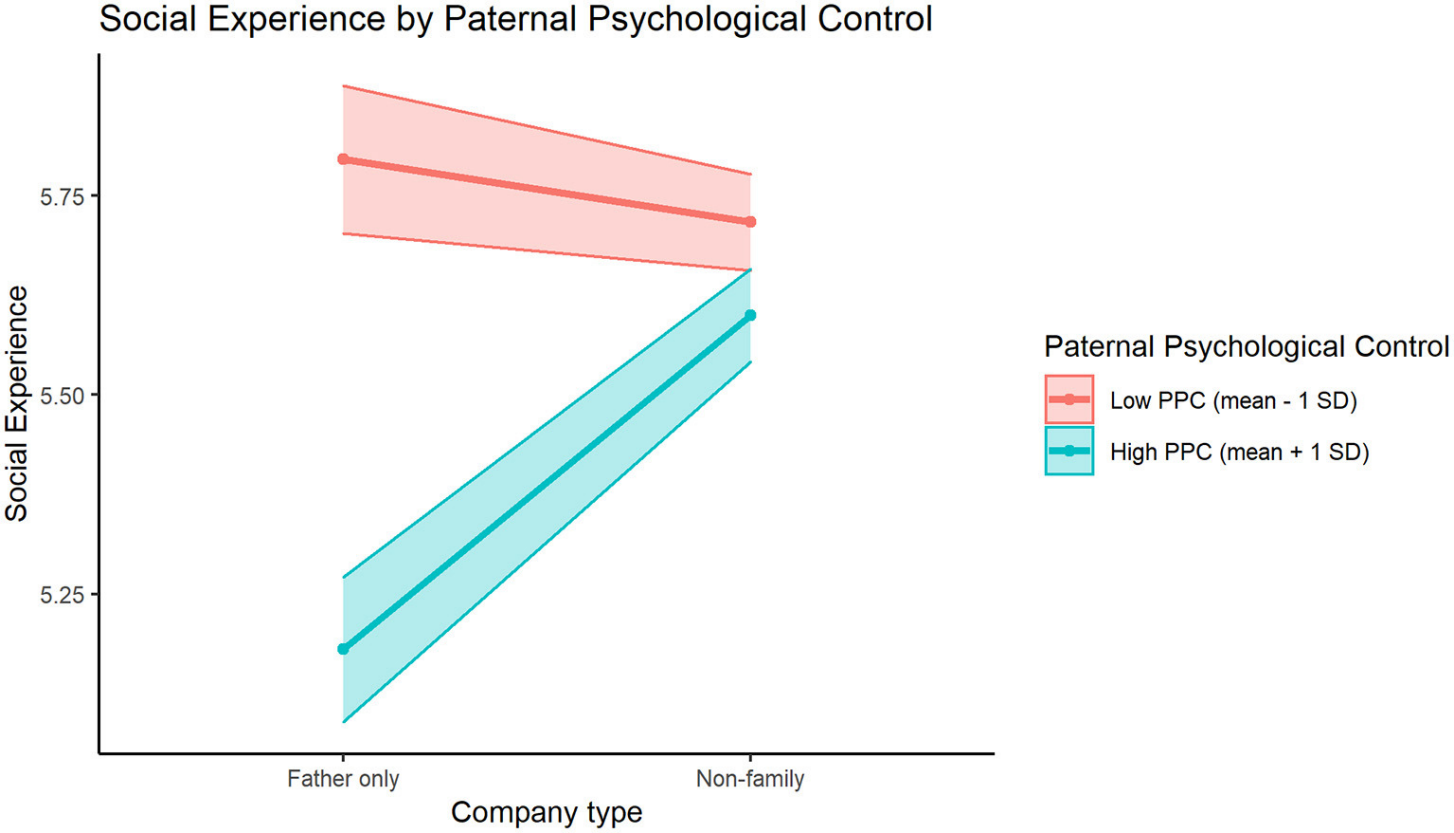
The significant interaction effect between PPC (Paternal Psychological Control) and paternal vs. non-family company in the prediction of mean social experience, for participants ±1 standard deviation of the Paternal Psychological Control mean (5.66).

The directions of these interaction effects are visualized in Figures 1–**3**. In Figure 1, we can see how participants with low or high levels of maternal responsiveness both report higher mean social experience quality when they are with their mother, compared to when they are with non-family members. This effect of higher social experience quality when with mother is slightly larger for those with higher levels of maternal responsiveness. Figure 2 illustrates how participants with low or high levels of paternal autonomy support have comparable estimated social experience quality when they are with their mother, and both groups report worse social experience quality when they are with non-family members. However, for those with lower levels of paternal autonomy support, social experience quality when with non-family members is lower (compared to those with higher paternal autonomy support levels). Finally, in Figure 3, we see how participants with higher levels of paternal psychological control had a higher estimated negative social experience quality when they were with their father compared to when with non-family members, while participants with low paternal psychological control levels seem to have comparable estimated social experience quality when they are with non-family members and when they are with their father.

### Path Model

**Figure 4:**
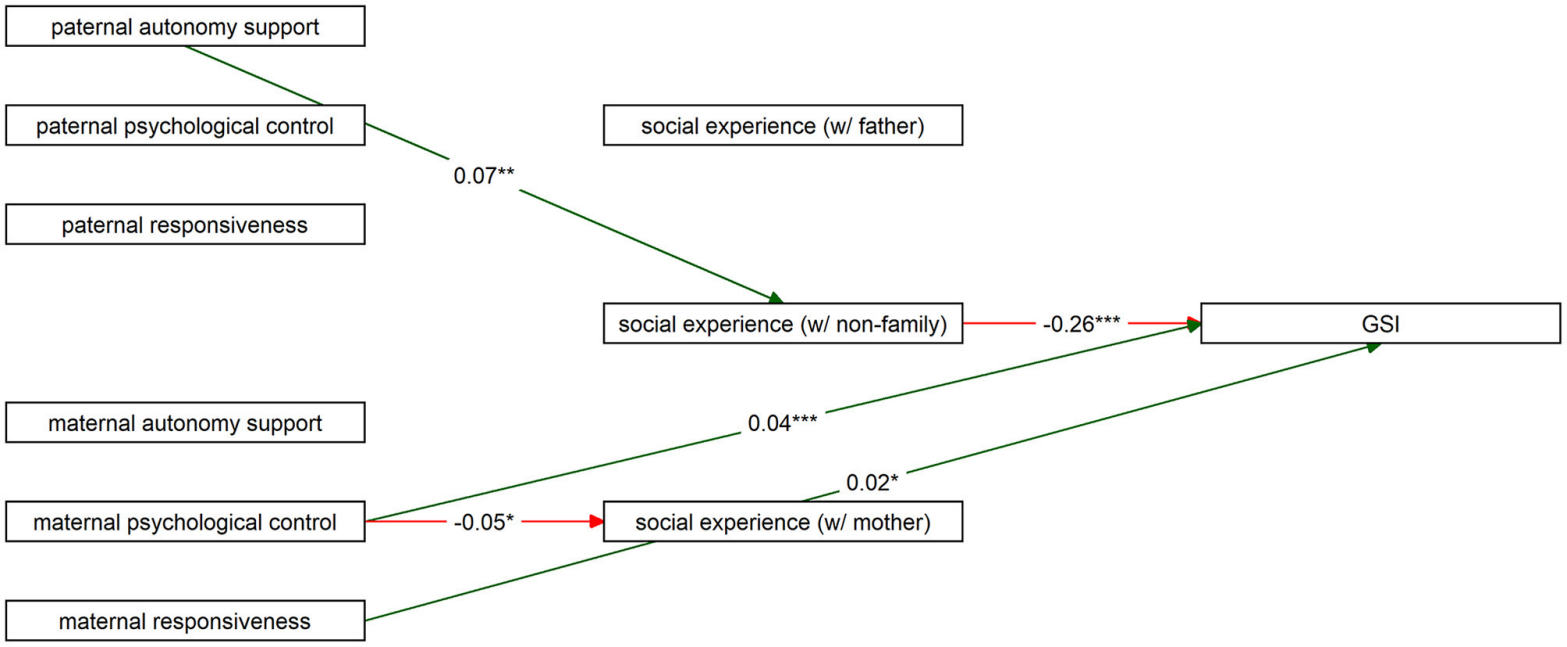
Visualization of comprehensive path model, with only the significant direct associations between variables displayed. Figure was constructed using the ‘*tidySEM'*-package (48). GSI, General Severity Index, or mean psychopathology. * *p* < 0.05; ** *p* < 0.01; *** *p* < 0.001.

To test the corrected associations between social experience in different companies, parenting styles and general psychopathology, we tested a path model that included these variables. The results of this path model are presented in Table 4. Fit statistics indicate extremely good fit of this model vs. the null model (*X*^2^ = 1016.82, *p* < 0.001; CFI = > 0.99; TFI > 0.99), and extremely good absolute fit (RMSEA 90% CI = [ < 0.001– < .001]; *p* <0.001; SRMR < 0.001). The statistically significant direct effects are visualized in Figure 4. Paternal autonomy support is a positive predictor of mean social experience when with non-family members; maternal psychological control is negatively associated with social experience when with mother only, and with general psychopathology levels; maternal responsiveness is positively associated with general psychopathology levels; and of the mediators, only mean social experience when with non-family members is negatively associated with general psychopathology levels.

**Table 4:** All standardized direct effects in the comprehensive, exploratory path model with parenting styles as six independent variables; three mediating variables of mean daily-life social experience; and one outcome variable of psychopathology.

**Direct effects**				
**Outcome**	**Predictor**	**B(SE)**	**95% CI**	***p***
GSI	Social experience-mother only	0.05 (0.04)	−0.03; 0.12	0.22
	Social experience-father only	0.00 (0.02)	−0.04; 0.05	0.90
	Social experience-non-family	−0.26 (0.04)	−0.34; −0.17	** <0.001**
	PAS	0.01 (0.01)	−0.01; 0.04	0.30
	PPC	0.02 (0.01)	−0.00; 0.04	0.09
	PR	−0.02 (0.01)	−0.04; 0.00	0.07
	MAS	0.00 (0.01)	−0.02; 0.03	0.71
	MPC	0.04 (0.01)	0.02; 0.06	** <0.001**
	MR	0.03 (0.01)	0.00; 0.05	***0.049***
Social experience-mother only	PAS	0.02 (0.03)	−0.03; 0.01	0.41
	PPC	0.02 (0.02)	−0.03; 0.01	0.46
	PR	0.02 (0.02)	−0.02; 0.06	0.38
	MAS	0.04 (0.03)	−0.02; 0.01	0.16
	MPC	−0.05 (0.02)	−0.09; −0.01	***0.013***
	MR	−0.01 (0.03)	−0.06; 0.05	0.82
Social experience-father only	PAS	0.07 (0.04)	−0.02; 0.15	0.13
	PPC	−0.05 (0.03)	−0.11; 0.02	0.17
	PR	0.04 (0.03)	−0.02; 0.09	0.25
	MAS	0.01 (0.04)	−0.07; 0.09	0.79
	MPC	−0.01 (0.03)	−0.07; 0.05	0.66
	MR	−0.03 (0.04)	−0.11; 0.05	0.45
Social experience-non-family	PAS	0.07 (0.02)	0.02; 0.11	**0.003**
	PPC	−0.02 (0.02)	−0.05; 0.02	0.29
	PR	−0.00 (0.02)	−0.04; 0.03	0.79
	MAS	0.01 (0.02)	−0.03; 0.05	0.69
	MPC	0.00 (0.02)	−0.03; 0.03	0.85
	MR	0.03 (0.02)	−0.01; 0.07	0.17

*PAS, Paternal Autonomy Support; PPC, Paternal Psychological Control; PR, Paternal Responsiveness; MAS, Maternal Autonomy Support; MPC, Paternal Psychological Control; MR, Maternal Responsiveness; GSI, General Severity Index, or mean psychopathology. P-values in bold italics indicate p < 0.05; p-values in bold only indicate p < 0.01*.

## Discussion

In this study, we found support for several of our hypotheses. We found support for maternal psychological control and responsiveness, and paternal autonomy support, specifically relating to the quality of adolescents' daily-life social interactions. When investigating the link between parenting styles and daily-life social experiences in different types of company, we saw a number of significant interaction effects: Maternal responsiveness and paternal psychological control were differentially associated with the experience of social interactions with parents. Paternal autonomy support was predictive of a more positive experience of social interactions with non-family members. This latter relationship was also significant in the comprehensive path model and was associated with psychopathology symptom levels. Moreover, this path model demonstrated how adolescents with generally more positive non-family (but not mother or father) interactions had lower levels of psychopathology symptoms.

The results of this study highlight the great relevance of altered social experiences—particularly with those outside the family—for understanding the impact of parenting styles on adolescents' (social) well-being. This study highlights the general and pervasive effects of maladaptive parenting styles, as parenting seems to broadly affect adolescents' interactions with parents and non-family members at the micro-level of everyday life. Moreover, the current study illustrates how this relationship could be relevant for the development of psychopathology, with a particular role for fathers and social experiences with those outside the family.

### Parenting Styles and Daily-Life Social Experiences

The first aim of this study was to identify those parenting styles that are particularly relevant for adolescents' experience of daily social interactions. We found maternal psychological control, maternal responsiveness and paternal autonomy support to be of specific relevance for social experience quality. The unique effect of these three different parenting styles aligns with the theoretical conception of these parenting styles as distinct influences on adolescents' psychosocial development (53).

Autonomy-supportive parenting arose as a particularly impactful factor, as it differentiated adolescents who experienced social interactions with non-family members as positive vs. negative. Parental autonomy support has been described as stimulating the volitional functioning of children by empathizing with children's point of view, allowing them to make decisions, and generally encouraging them to take initiative at their own pace (14, 35). While the adaptive outcomes of parental autonomy support are largely conceptualized as adjustment in terms of academic competencies, there is also evidence for autonomy support fostering children's social competence (54). In this study, we focused on the experienced quality of social interactions rather than the competencies to engage in social behaviors. However, it might be that social competence is a mediating factor here, as the reported positive social interactions with non-family members for those high in paternal autonomy support might be explained best by autonomy-support-driven increased social competence.

Of note is that, in previous work on a comparable sample of Flemish adolescents, *maternal* rather than *paternal* autonomy support was found to contribute mostly to social competence (54). As our sample was similar, the difference with our study—where we report a unique impact of paternal rather than maternal autonomy support—might reflect a change in mothers' and fathers' roles in the 15 years between these studies. An increased unique relevance of fathers for children's development has been reported in other recent studies as well (55). What these findings indicate is that fathers and mothers have unique, complementary roles. This notion has also been emphasized by attachment researchers (56, 57), as early attachment and parenting research has not sufficiently considered the distinct roles of mother vs. father.

Interaction effects revealed social effects of maternal responsiveness and paternal psychological control—however, these effects seemed mostly related to differential experiences of parent rather than non-family interactions. Parental responsiveness is defined in terms of emotional support provision and expressed affection (2), and as such, it is not entirely unexpected that adolescents with more perceived maternal responsiveness also experience interactions with their mother as more positive. However, while this effect of responsiveness on social experiences is stronger for interactions with mothers, it seemed that those with more responsive mothers also perceived their non-family interactions as more positive.

Psychological control is usually regarded as a particularly maladaptive parenting style (1, 8), and we found a significant direct effect of maternal psychological control on the mean quality of all social interactions. Also, interaction effects indicated that for those with high levels of psychological control, the quality of social experiences with their father were worse than interactions with non-family members. At the same time, high levels of psychological control did not seem associated with worse experiences of non-family interactions. This finding is in contrast to the expectation that psychological control might have a unique detrimental effect across different types of social interactions and might, as such, drive the development of psychopathology.

### The Mediating Role of Social Experiences in Predicting Psychopathology

Although the mediation of the relationship between parenting styles and psychopathology by altered daily-life social experiences is implied in most theories of developmental psychopathology (58), it has scarcely been explored at the level of daily life. The path model in this study, however, explicitly illustrates this tacit assumption, and suggests a potential mediating role for altered social experiences with non-family members specifically—as the only mean social experience variable that significantly predicted psychopathology levels was the variable representing the non-family member social experiences.

The particular relevance of non-family interactions is consistent with theories on adolescent social development, which emphasize a shift from parents to peers in the transition toward adulthood (59). The current findings add to this theoretical notion by demonstrating how positive social experiences outside of the family actually represent an important correlate of psychopathology symptom development. This does not negate the value of good parent-child relationships, as we also see how social experiences outside the family might be partially shaped by experiences with parents—notably, by autonomy supportive parenting. However, the findings of this study emphasize a particular function of adaptive parenting, as it can prepare adolescents for interacting with people outside of the family with an increased sense of security. Although the current study precludes causality claims, in light of the larger developmental psychopathology body of evidence, it is exactly this greater sense of security that seems crucial for protecting against the development of psychiatric distress.

### Strengths and Limitations

This study has several considerable strengths. First, we were able to draw on one of the largest experience-sampling datasets available, which allowed for reliable and ecologically valid estimates of the experiences of adolescents' social interactions. The manner in which social interactions were measured in this study is relatively unique, assessing adolescents' experiences of social interactions at the moment that they engage in them—rather than some time afterwards. Second, this study was registered before data access (but following data collection), and all code and analyses are available online. This contributes to transparency, reproducibility, and replicability efforts which are increasingly recognized as priorities in both clinical psychology and experience-sampling research (60–62).

Despite these strengths, the results of this study should be interpreted in light of its limitations. First, compliance to the experience sampling protocol was relatively low, compared to other experience sampling studies, which usually involve compliance levels between 70 and 80% (63). This low compliance might also drive the relatively few endorsements of our outcome variable of social interactions with solely mother or father. One likely cause for the low compliance is the lack of incentivization in this study, as many ESM studies pay participants per completed questionnaire. We did not use compliance-contingent incentivization in this study, as we feared that this might increase careless responding. Still, additional research is required to test this assumption. Another potential cause for the low compliance is the relatively short response delay of 90 s to respond to each ESM questionnaire. While allowing for longer response delays might yield increased compliance, it also undermines one of the key strengths of ESM, that is, to measure as ‘in the moment' as possible. Recent research suggests that longer response delays in ESM studies do in fact lead to qualitatively different responses (64), thereby underscoring the importance of short response delays. Furthermore, the path model was based on cross-sectional data, and does not allow us to make inferences on the temporal ordering or causality of the investigated associations (27, 28). It is highly likely that psychopathology levels also affect both perceptions of parenting and daily-life social experiences. In addition—as widely recognized by developmental psychologists—the parent-child relationship is bidirectional in nature, as parenting styles are also affected by children's behaviors (13, 65). Therefore, the path model presented here should be considered as exploratory and descriptive rather than confirmatory. Future work is needed to shed additional light on the sequence of events in the presented mediation model.

### Implications and Future Directions

The results of this study speak to potentially impactful implications. Notably, while much previous research has focused on the importance of adaptive maternal parenting, these results emphasize how fathers play a unique role in the socialization and development of children and adolescents as well. Paternal autonomy supportive parenting distinctly related to the experience of day-to-day social interactions with non-family members, and through this association, also with psychopathology levels. If this finding proves robust following future replication, parenting intervention programs should ensure that they are inclusive of both fathers and mothers, and stimulate autonomy supportive parenting. Additionally, as the quality of interactions with non-family members appeared uniquely associated with psychopathology symptoms, accurate assessment of social experiences in daily-life might provide a valuable focus for mental health prevention and treatment programs. This may take the form of, for example, ecological momentary interventions (EMIs) that aim to explicitly target daily-live processes for the treatment of early psychopathology (66, 67).

Before being able to translate the current exploratory findings into concrete recommendations, however, several questions need to be answered. Primarily, as this study focused mainly on assessing general social experiences to predict general psychopathology, a further ‘unpacking' of both these variables and of parenting styles and behaviors allows for the identification of more specific mediation processes in the relationship between parenting psychopathology. For example, autonomy supportive parenting is believed to instill a general sense of volitional functioning in children, making them more likely to approach peers, which in turn, might foster friendships and protect against the development of psychopathology (54). In addition, our study did not assess parenting behaviors with ESM, although an increasing number of studies have demonstrated the feasibility and usefulness of focusing on more micro-level parent-child processes (68, 69). Research that considers both predictors and outcomes at the level of daily life (e.g., from both child and parent) not only allows for examining these micro-level processes in more detail, but also allow for assessing individual variation in within-person or within-family processes (70). Future work could test micro-level hypotheses with increasing accuracy—thereby generating essential knowledge of the adaptive and maladaptive processes that play out in adolescents' day-to-day interactions.

## Data Availability Statement

The datasets presented in this article are not readily available because data are only available through an abstract submission process, as described in Kirtley et al. (29) (for more information, see https://psyarxiv.com/jp2fk/). Requests to access the datasets should be directed to olivia.kirtley@kuleuven.be.

## Ethics Statement

The studies involving human participants were reviewed and approved by Ethic Committee Research UZ/KU Leuven S61395. Written informed consent to participate in this study was provided by the participants' legal guardian/next of kin.

## Author Contributions

RA, OK, MS, and IM-G were all involved in the conceptualization of this study. RA and GL designed the methodology. RA performed data analysis. RA, NH, KH, AH, and AL were involved in data collection. RA wrote the original draft of the manuscript. OK, MS, NH, KH, AH, AL, GL, and IM-G were all involved in subsequent review and editing of this manuscript. RA constructed the figures. OK and IM-G supervised the project leading to this research, with OK managing and coordinating the research activity. IM-G acquired the funding for this research. All authors contributed to the article and approved the submitted version.

## Conflict of Interest

The authors declare that the research was conducted in the absence of any commercial or financial relationships that could be construed as a potential conflict of interest.

## Publisher's Note

All claims expressed in this article are solely those of the authors and do not necessarily represent those of their affiliated organizations, or those of the publisher, the editors and the reviewers. Any product that may be evaluated in this article, or claim that may be made by its manufacturer, is not guaranteed or endorsed by the publisher.
