# Supplementary material for: Daily-Life Social Experiences as a Potential Mediator of the Relationship Between Parenting and Psychopathology in Adolescence
Source: Front Psychiatry. 2021 Aug 5;12:697127. doi: 10.3389/fpsyt.2021.697127 (PMC8374596; doi:10.3389/fpsyt.2021.697127)
Supplement: Supplementary file 1 [file Data_Sheet_1.docx]

# Supplement – Experience Sampling Method Questionnaire, SIGMA Wave I

The SIGMA ESM questionnaire is also publicly available in the ESM Item Repository (www.esmitemrepository.com; Kirtley et al., 2020; dataset: ‘sigma’).

If not multiple choice (MC), items will be rated on a 7-point Likert scale, ranging from 1 (not at all) to 7 (very much).

## Beep questionnaire throughout the day

### Positive and negative affect

1) Ik voel me vrolijk (I feel cheerful)

2) Ik voel me geïrriteerd (I feel irritated)

3) Ik voel me angstig (I feel anxious)

4) Ik voel me tevreden (I feel satisfied)

5) Ik voel me onzeker (I feel insecure)

6) Ik voel me eenzaam (I feel lonely)

7) Ik voel me ontspannen (I feel relaxed)

8) Ik voel me wantrouwig (I feel paranoid)

9) Ik voel me verdrietig (I feel sad)

10) Ik voel me gestresst (I feel stressed)

11) Ik voel me rusteloos (I feel restless)

12) Over het algemeen gezien voel ik me nu goed (In general I feel well at the moment)

13) Ik kan me goed concentreren (I can concentrate well)

14) Ik voel me zeker van mezelf (How much self-confidence do you have?)

### Identity

15) Ik kan mezelf zijn (I can be myself)

16) Ik twijfel wie ik ben (I doubt who I am)

17) Ik weet waar ik voor sta (I know where I stand for)

### Social context

18) Met wie ben ik? (Who is with me?)

MC: Familie thuis (gezin) - andere familie (other family) - vriend(en) (friends) – andere leeftijdsgenoten (other peers) - leraar (teacher) - onbekende mensen (unknown people/others) - niemand (nobody)

*If alone:*

19) Ik vind het fijn om alleen te zijn (I find being alone pleasant)

20) Ik wil alleen zijn (I want to be alone)

21) Ik voel me buitengesloten (I feel an outsider)

22) Ik wil liever met anderen zijn (I prefer to have company)

*If in company:*

19) We zijn samen iets aan het doen (praten, leren, gamen, etc.) (We’re interacting)

20) Ik voel me op mijn gemak in dit gezelschap (I feel comfortable in this company)

21) Ik voel me gewaardeerd door dit gezelschap (I feel valued in this company)

22) Ik hoor erbij

23) Ik zou liever alleen zijn (I prefer to be alone)

24) Ik ben virtueel in contact met anderen (I’m interacting with others online)

MC: Ja – Nee

*If yes:*

25) We zijn samen iets aan het doen (We’re interacting)

26) Ik voel me op mijn gemak in dit gezelschap (I feel comfortable in this company)

27) Ik voel me gewaardeerd door dit gezelschap (I feel valued in this company)

28) Ik hoor erbij (I belong to the people I’m interacting with)

*If no:*

25) Ik zou graag virtueel in contact zijn met anderen (I would like to be contact with others, virtually)

26) Hier heb ik zelf voor gekozen (This is my choice)

27) Ik voel me buitengesloten (I feel an outsider)

### Physical context

29) Wat ben je aan het doen? (What are you doing?)

MC: vrije tijd actief (sporten, uitgaan) (leisure active) – vrije tijd passief (tv kijken, lezen) (leisure passive) – school/werk (school/job) – huishoudelijke taken (chores) - onderweg (on my way) - persoonlijke hygiene (wassen/tanden poetsen/aankleden) (personal hygiene) – iets anders (other) - niets (nothing)

30) Dit vind ik leuk (I like this activity)

31) Ik zou liever iets anders doen (I would rather do something else)

32) Dit is moeilijk voor mij (This is difficult for me)

33) Ik kan dit goed (I can do this well)

34) Ik ben actief bezig (I’m engaged)

35) Waar ben je? (Where are you?)

MC: op school (at school) - thuis (at home) - bij vriend(en) (at friends’ place) - bij andere familie dan thuis (other family than home) - op de fiets/in de auto/in de bus/in de trein (public transport) - ergens anders binnen (other indoors) – ergens anders buiten (other outdoors)

### Control items

36) Ik ben moe (I am tired)

37) Ik heb honger (I am hungry)

38) Ik voel me niet lekker (I feel physically unwell)

39) Ik heb pijn (I am in pain)

### Anticipation of pleasure

Denk aan de belangrijkste situatie voor jou in de komende 2 uur (Think about the most important situation for you in the next 2 hours)

40) Hoeveel zin heb je in deze situatie? (How much are you looking forward to this situation?)

41) Deze situatie hoort bij de categorie: (This situation belongs tot he following category:)

MC: lichamelijke inspanning (physical activity) – school/werk (school/job) – actieve ontspanning (active relaxation) – passieve ontspanning (passive relaxation) – slapen (sleeping) – iets anders (something else) – eten/drinken (eating/drinking) – niets (nothing)

### Suicidal ideation/self-harm

Sinds de vorige beep: (Since the last beep:)

42) Heb je eraan gedacht om jezelf te verwonden? (Have you considered harming yourself?)

*If rated with 2-7:**

43) In welke mate had je tijdens deze gedachten over zelfverwonding de wens om je leven te

beëindigen? (During these self-harm thoughts, how much did you wish to end your life?)

44) Heb je hiernaar gehandeld door jezelf opzettelijk te verwonden, pijn te doen of te vergiftigen?

(Have you actually harmed yourself on purpose?)

MC: Ja – Nee (Yes – No)

### Substance use

45) Sinds de vorige beep heb ik het volgende gebruikt: (Since the last beep I’ve used:)

MC: niets (nothing); medicatie (medication); alcohol (alcohol); sigaretten (cigarettes);

cannabis (cannabis); andere drugs (other drugs)

46) Deze beep stoorde me (This beep disturbed me)

*** Pop-up will show if participants report thoughts on suicide and/or self-harm:**

Het lijkt erop dat je het op dit moment misschien moeilijk hebt. Onthoud dat als je anoniem met iemand wilt praten over hoe je je voelt, dat kan op:

Awel op 102, tussen 16u - 22u; Tele-Onthaal op 106, 24 uur per dag; Zelfmoordlijn, 1813, 24 uur per dag.

Het kan natuurlijk ook altijd goed zijn om met iemand te praten die dicht bij je staat, zoals een vriend, ouder, of leraar.

Mocht er een noodgeval zijn, en je hebt direct medische hulp nodig, bel dan gelijk 112.

**English translation of pop-up:**

It looks like you’re having a difficult time at the moment. Remember that if you would like to talk to someone anonymously about how you are feeling, you can: AWEL on 102, between 16:00 – 22:00; Tele-Onthaal on 106, 24 hours a day; the Flemish suicide crisis line 1813, 24 hours a day. Of course, it’s always good if you can talk to someone close to you, like a friend, parent or teacher. If you are in an emergency situation and you need immediate medical help, call 112.
