# Supplementary material for: Daily-Life Social Experiences as a Potential Mediator of the Relationship Between Parenting and Psychopathology in Adolescence
Source: Front Psychiatry. 2021 Aug 5;12:697127. doi: 10.3389/fpsyt.2021.697127 (PMC8374596; doi:10.3389/fpsyt.2021.697127)
Supplement: Supplementary file 2 [file Data_Sheet_2.docx]

## Transparent Changes to postregistration for ‘Daily-life Social Experiences as a Mediator of the Relationship Between Parenting and Psychopathology in Adolescence’

This study was officially postregistered on the Open Science Framework on August 21^st^, 2019, with an addendum to the registration (detailing the exploratory mediation analysis) added on August 23^rd^, 2019. Both registrations are available through <https://bit.ly/3wH3LPE> and <https://bit.ly/2R9Cvsu>. In error, the original registration had previously been uploaded as a file to the OSF-page for this project on June 26th, 2019, instead of as a registration. This file upload occurred prior to data access and analysis. Data for this study were accessed on June 28^th^ – thus, prior to the official registration, but following the initial upload of the registration to the OSF-page. Note that the original registration dated June 26^th^ is the same as the official registration dated August 21^st^.

In this document, we transparently describe all changes that we made to our analysis plan following study registration, i.e. deviations from our original postregistration All code relevant to this study is uploaded to the OSF-page of this project (<https://osf.io/vz4hy/>

- In the registration for this study, we originally planned to exclude participants from the study if they had completed fewer than 30% of all ESM questionnaires. This was based on common practice in many ESM studies and was first recommended ESM research guidelines from 1995 (1). However, the rationale for this 30%-exclusion rule has been subsequently questioned, as omitting participants from analyses might bias the results. (2). Therefore, for our main analysis, we decided to include all participants with valid ESM data, and to perform a sensitivity analysis applying the 30%-rule we originally specified.
- In the analysis section of our registration, we described tests of whether paternal parenting styles predict only paternal social experiences, and whether maternal parenting styles predict only maternal social experiences (all as compared to non-family interaction experiences). Afterwards, we decided against this, and to test the interactions between each parenting variable and both the ‘with mother’ and ‘with father’ categories of the company variables. The reason for this was because, to assess the specificity of same-sex versus different-sex parent effects, associations with parents of both genders need to be considered in the analysis.
- In the analysis section of our registration, we planned to conduct post-hoc Wald-type tests, to investigate whether the association between each parenting style and social experience was significantly different when social experience pertained to a parent versus non-family members. However, these Wald-tests were unnecessary, as this difference in effects is directly tested with the included interaction effects.
- In the registration, we noted that we would apply Simes’ multiple comparison correction (3) to our confirmatory analysis results. However, we later realized this was inappropriate, as such a multiple comparison correction requires independent tests (which we do not have). Moreover, our confirmatory analysis involves multiple predictors that are all included in the same model, meaning they are already corrected, and do not require the strict multiple comparison correction that we had planned.
- The addendum to the registration describes a mediation analysis, testing the indirect effects of parenting styles on psychopathology through altered social experiences. Based on later discussions within the team and among external researchers, we revisited the literature (4,5) and decided it was inappropriate to test for mediation effects using the cross-sectional data from the current study. . Therefore, for this mediation analysis, we made some changes, i.e. to only test the cross-sectional associations between all relevant variables that would be expected to be associated with each other following the mediation model. We used the ‘lavaan’-package to test the full hypothesized path model with six predictors, three mediator variables, and one outcome, thereby obtaining the corrected coefficients for the direct effects. Indirect effects were not estimated in this comprehensive path model.

## References

1. Delespaul PAEG. Assessing schizophrenia in daily life : the experience sampling method. UPM, Universitaire Pers Maastricht; 1995. 374 p.

2. Jacobson NC. Compliance thresholds in intensive longitudinal data: Worse than listwise deletion: Call for action [Symposium] [Internet]. Society for Ambulatory Assessment Conference, Melbourne, Australia. 2020. Available from: http://www.nicholasjacobson.com/files/talks/SAA2020_Compliance_Thresholds.pdf

3. Simes RJ. An improved Bonferoni procedure for multiple tests of significance. Biometrika [Internet]. 1986;73(3):751–4. Available from: https://academic.oup.com/biomet/article-abstract/73/3/751/250538

4. Maxwell SE, Cole DA. Bias in cross-sectional analyses of longitudinal mediation. Psychol Methods [Internet]. 2007 Mar;12(1):23–44. Available from: /record/2007-03329-002

5. Cole DA, Maxwell SE. Testing Mediational Models with Longitudinal Data: Questions and Tips in the Use of Structural Equation Modeling [Internet]. Vol. 112, Journal of Abnormal Psychology. American Psychological Association Inc.; 2003. p. 558–77. Available from: /record/2003-10098-004
